# Three New Species of *Aphelinus* (Hymenoptera: Aphelinidae) from China, with a Note on the *japonicus* Group †

**DOI:** 10.3390/insects16121205

**Published:** 2025-11-27

**Authors:** Zhigang Dong, Junqing Ge, Jian Huang, Zhuhong Wang

**Affiliations:** 1 State Key Laboratory of Agricultural and Forestry Biosecurity, College of Plant Protection, Fujian Agriculture and Forestry University, Fuzhou 350002, China; d18235546505@163.com (Z.D.); jhuang1234@126.com (J.H.); 2 Institute of Biotechnology, Fujian Academy of Agricultural Sciences, Fuzhou 350003, China; jqge@163.com

**Keywords:** Aphelinidae, *Aphelinus*, aphid parasitoids, biocontrol, *cox1*, taxonomy

## Abstract

In this paper, three new species of *Aphelinus* (Hymenoptera: Aphelinidae), *Aphelinus jinshanensis* Wang & Huang, **sp.n.**, *Aphelinus albimaculatus* Wang & Huang, **sp.n.** and *Aphelinus varius* Wang & Huang, **sp.n.** are described and illustrated. Additionally, the *japonicus* group of *Aphelinus* is reviewed. The *japonicus* group is differentiated from all the known groups of these three subgenera by having a 3-segmented flagellum in the male. It is characterized by the following combination of features: antenna 6-segmented in the female, and 5-segmented in the male; body yellow, partly dark brown to black; the numerous setae on mid-lobe not arranged in symmetry; ovipositor short, and hypopygium not reaching to the apex of the gaster. *Aphelinus varius* Wang & Huang, **sp.n.** described here represents the second species assigned to the *japonicus* group. An identification key to the species of this group is provided. The relationships among these three new species are analyzed using a maximum likelihood phylogenetic tree based on *cox1* sequences. While the evolutionary relationships of some species or groups remain uncertain due to weak statistical support for the relevant branches.

## Simple Summary

Species of *Aphelinus* (Hymenoptera: Aphelinidae) are obligate primary parasitoids of aphids (Hemiptera: Aphidoidea) and some have been successfully used in the biological control of aphid pests. *Aphelinus* is a moderately diverse genus within the family Aphelinidae, currently comprising 102 described species worldwide, with 35 species recorded in China. This genus is divided into three subgenera: *Aphelinus*, *Indaphelinus* and *Mesidia*, and further classified into three species groups in *Mesidia*, four species groups in *Aphelinus* and one species group in unknown subgenera. Among these, the *japonicus* group is particularly distinctive due to the presence of a 3-segmented flagellum in males, setting it apart from the three known subgenera; however, other species of *Aphelinus* possess a 4-segmented flagellum in both males and females. In this paper, we describe three new species of *Aphelinus* and summarize knowledge of the *japonicus* group.

## 1. Introduction

Species of the genus *Aphelinus* (Hymenoptera: Chalcidoidea: Aphelinidae) are primary parasitoids of aphids (Hemiptera: Aphidoidea) [1,2]. As important parasitoid natural enemies, some species of *Aphelinus* have been utilized effectively for the biological control of aphid pests, such as the successful use of *A. mali* (Haldeman) for the biological control of *Eriosoma lanigerum* (Hausmann), the apple wooly aphid [3,4]. In Europe, *Aphelinus abdominalis* Dalman is extensively applied for the control of the potato aphid, *Macrosiphum euphorbiae* (Thomas) and the greenhouse potato aphid, *Aulacorthum solani* Kaltenbach [5], and in the United States, Japan and other countries, *Aphelinus varipes* is considered as a potential biological agent against the wheat aphid, *Diuraphis noxia* (Mordwilko) and the soybean aphid, *Aphis glycines* Matsumura [6,7].

*Aphelinus* is moderately diverse within the family Aphelinidae [2,8]. So far, it comprises 102 described species worldwide [9], among which 35 species are recorded from China [2,10,11,12,13,14,15,16,17,18]. *Aphelinus* is classified into three subgenera: *Aphelinus*, *Indaphelinus* and *Mesidia* [8,19]. In two subgenera, the subgenus *Aphelinus* (*Aphelinus*) includes four species groups: *abdominalis*, *asychis*, *mali* and *nepalensis*, and the subgenus *Aphelinus* (*Mesidia*) contains three species groups: *argiope*, *subflavescens* and *automatus* [8]. The *japonicus* group is not assigned to any of these three established subgenera [20].

Hayat (1991) redescribed *Aphelinus japonicus* Ashmead known only from the female from Japan and noted that it differs from all previously known species of *Aphelinus* by the partly dark color of the thorax, the numerous setae on the mid-lobe not arranged in symmetry, the short ovipositor, and the hypopygium not reaching to the apex of the gaster [20]. Based on the above-mentioned characteristics, Hayat (1991) established the *japonicus* group, which is distinct from the three known subgenera and comprises solely *A. japonicus* [20]. Subsequently, Hayat (1994) [21] first described the male of *A. japonicus* collected by C.M. Yoshimoto from Japan in 1980 [21]. The male is rather unique within *Aphelinus* in possessing a 3-segmented flagellum, with F1 being anelliform and the following two segments being elongate and setose [21]. In contrast, all other species of *Aphelinus* possess a 4-segmented flagellum in both males and females.

To date, there are three species of *Aphelinus* characterized by antennal variation in the male: *A. argiope*, *A. asychis* and *A. japonicus* [8,21]. Among these, *A. japonicus* is unique with a 3-segmented flagellum. Furthermore, *A. varius* Wang & Huang, **sp.n.** described below is close to *A. japonicus* characterized by a 3-segmented flagellum with the anelliform F1 and the other two long and setose segments in the male, and this new species is assigned to the *japonicus* group.

In this study, three new species, *Aphelinus jinshanensis* Wang & Huang, **sp.n.**, *Aphelinus albimaculatus* Wang & Huang, **sp.n.**, and *Aphelinus varius* Wang & Huang, **sp.n.** are described and illustrated. A maximum likelihood phylogenetic tree is constructed based on *cox1* sequences to analyze the phylogenetic relationships among some species within *Aphelinus*. The *japonicus* group with special antennal configuration in males is summarized, and an identification key to species is provided.

## 2. Materials and Methods

### 2.1. Collection of Parasitoids

Aphid specimens were partly collected from *Podocarpus macrophyllus* in Fujian Province, and Bamboo in Beijing, and observed in the laboratory for the emergence of parasitoids. Additionally, some specimens were obtained using a sweep net. *Aphelinus* parasitoids reared from these aphids were preserved in 100% ethanol after emergence.

### 2.2. Photograph and Slides of Parasitoids

Prior to slide mounting, the body color of each specimen was described and photographed. Specimens were mounted on slides according to the method described by Noyes (1982) [22]. Specimens were photographed using a Nikon DS-Ri2 camera, with NIS-Elements D v4.40 software, attached to a Nikon SMZ18 microscope (Nikon Corporation, Shanghai, China). Slide-mounted specimens were photographed by a Nikon Ni microscope (Nikon Corporation, Shanghai, China) with the same camera and software. Body length measurements were obtained from specimens before slide mounting, and all other measurements were taken from slide-mounted specimens. Type material and the other specimens examined in the study are deposited in the College of Plant Protection, Fujian Agriculture and Forestry University, Fuzhou, China (FAFU).

### 2.3. DNA Sequencing

Genomic DNA was extracted based on the method of Polaszek et al. (2013) [23] using the DNeasy Blood & Tissue Kit (Qiagen, Hilden, Germany), and the extracted specimens remained intact. These specimens were then permanently preserved in Canadian balsam. Primer sequences and cycling conditions are given in Table 1 [24]. Polymerase chain reaction (PCR) was performed on 2 μL genomic DNA extract. Each 25 μL reaction mix contained 12.5 μL 2 × Taq PCR MasterMix II, 1 μL forward primer, 1 μL reverse primer, 2 μL genomic DNA extract and 8.5 μL ddH_2_O. DNA was sequenced at Sangon Biotech (Shanghai, China) using the same primers used for the PCR reaction product. Forward and reverse sequences were assembled and edited using DNAMAN version 9.0 and submitted to GenBank (GenBank notes see Table 2).

### 2.4. Phylogenetic Reconstruction

The species and their GenBank accession numbers used in phylogenetic analysis are shown in Table 2. Using the partial sequences determined in this paper and downloaded from NCBI-GenBank, the maximum likelihood phylogenetic tree was constructed with one *Coccophagus* species (Aphelinidae) as the outgroup. PhyloSuite v1.2.3 was used to extract *cox1* gene [25]. The FFT-NS-1 algorithm in MAFFT v7.313 was used to compared the *cox1* genes of these 17 species [26]. ModelFinder v2.2.0 [27] was used to select the optimal evolution model and the best model of BIC was Tim + F + I + I + R2. Finally, based on the best model of BIC, the maximum likelihood phylogenetic tree was constructed using IQ-TREE v1.6.8 with 1000 standard bootstrapping replicates [28]. The phylogenetic tree was edited in ITOL v7.2.2 (https://itol.embl.de) (accessed on 22 September 2025).

### 2.5. Terminology, Morphological Measurement and Abbreviations

Terminology and morphological measurement follow Huang (1994) [2] with some modification, and the following abbreviations are used: F1, F2, etc. = antennal funicle segments 1, 2, etc.; T1, T2, etc. = gastral tergites 1, 2, etc.

Abbreviations for depositories are as follows:

FAFU College of Plant Protection, Fujian Agriculture and Forestry University, Fuzhou, Fujian, China.

USNM United States National Museum of Natural History, Washington DC, USA.

## 3. Results

### 3.1. Species Accounts

#### 3.1.1. *Aphelinus jinshanensis* Wang & Huang, sp.n. (Figure 1)

*Diagnosis*. *Aphelinus jinshanensis*, **sp.n.** belongs to the subgenus *Aphelinus* (*Aphelinus*) and resembles *A. sanborniae*, but can be distinguished from the latter by: antennae with scape largely dark brown, tip of scape and other segments pale brown (*A. sanborniae*: antennae with flagellum pale orange yellow); fore leg with coxae, femur, the basal 1/2 of tibia dark brown, other pale yellow brown; mid leg with coxae, femur, tibia dark brown, other pale yellow brown; hind leg with coxae, tibia, basitarsus dark brown, femur pale yellow, tarsus pale yellow brown (*A. sanborniae*: legs black, hind leg with femur pale yellow, except tip of all tarsus pale yellow); fore wing with slight brown infuscation and with one complete line of 11 setae and 3–4 incomplete lines of 11 setae basal to the linea calva (*A. sanborniae*: fore wing hyaline, with one line setae and extra of 3–4 setae basal to the linea calva).

*Aphelinus jinshanensis*, **sp.n.** resembles *A. lusitanicus*, but can be distinguished from the latter by: fore leg with coxae, femur, the basal 1/2 of tibia dark brown, remainder pale yellow brown; mid leg with coxae, femur, tibia dark brown, remainder pale yellow brown; hind leg with coxae, tibia, basitarsus dark brown, femur pale yellow, tarsus pale yellow brown (*A. lusitanicus*: legs brown, only tip of tibia and basitarsus brown yellow); fore wing with slight brown infuscation and with one complete line of 11 setae and 3–4 incomplete lines of 11 setae basal to the linea calva (*A. lusitanicus*: with 5–6 lines of 60–64 setae).

*Description*. **Female**. Body length 0.57–0.65 mm. **Color**. Body black; head with eyes dark red brown; antennae brown yellow; wing hyaline; fore leg with coxae black brown, the basal 1/2 of trochanter, femur, tibia pale brown, distal 1/2 of trochanter, tarsus pale yellow, the last tarsal segments slightly dark; mid leg with coxae, femur, tibia black brown, trochanter, tarsus pale yellow, the last tarsal segments slightly dark, hind leg with coxae, tibia black brown, femur white, tarsus pale yellow brown, basitarsus and the last tarsal segments slightly dark. **Head**. Head 1.18× as broad as high in frontal view, about as broad as mesosoma; frontovertex 0.46× head width; mandible with two acute teeth; antennae with scape 4.42× as long as broad, pedicel 1.92× as long as broad, F1 annular, F2 1.69× as broad as long, F3 trapezoid, 1.26× as broad as long, club 1.88× as long as broad. **Mesosoma**. Mesonotum with fine reticulate sculpture, mid lobe of mesoscutum with 45–50 fine setae and 2 pairs of long setae, side lobes each with 1 long and 1 short setae; mesoscutellum with 2 pairs of long setae. **Fore wing**. 2.20× as long as broad; costal cell with 3 rows of setae, costal cell as long as marginal vein; submarginal vein bearing 6–7 setae, marginal vein bearing 8–9 setae along anterior margin, stigmal vein short; one complete line of 12 setae, and 3–4 incomplete lines of 11 setae basal to the linea calva; wing disc with dense setae. **Leg.** Mesotibial spur 1.23× mesobasitarsus; tarsal formula 5-5-5. **Metasoma**. 1.46× as long as mesosoma; ovipositor located basally at basal 0.5× of metasoma, 1.16× as long as mesotibia, third valvula 0.36× length of ovipositor.

**Male**. Unknown.

*Host*. Unknown.

*Distribution*. China (Fujian).

*Etymology*. The new species is named after the collecting locality, Jinshan.

Material. **Holotype** ♀, No.Y37, by sweep net. China: Fujian, Fuzhou, Fujian Agriculture and Forestry University, Jinshan campus, 13 November 2023, coll. Zhigang Dong (FAFU).

**Figure 1:**
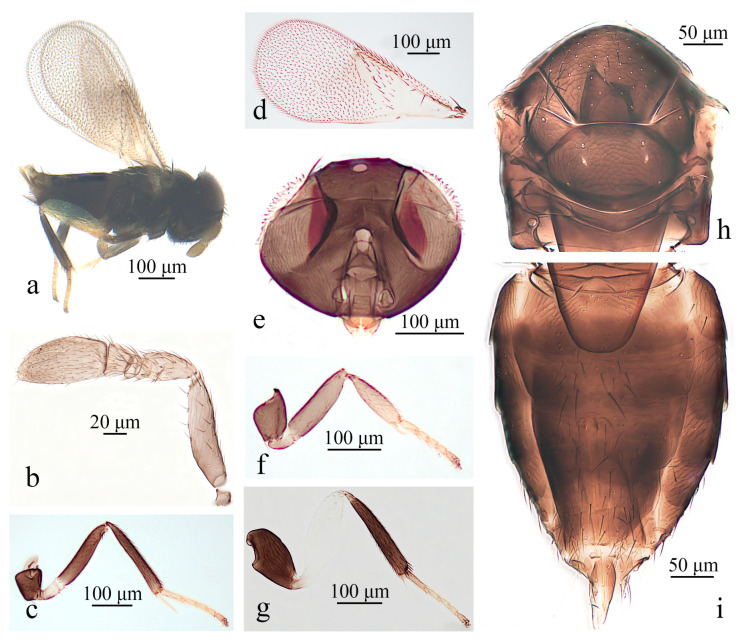
*Aphelinus jinshanensis*, **sp.n**., female. (**a**) Adult in lateral view; (**b**) antenna; (**c**) mid leg; (**d**) fore wing; (**e**) head; (**f**) fore leg; (**g**) hind leg; (**h**) mesosoma; (**i**) metasoma.

#### 3.1.2. *Aphelinus albimaculatus* Wang & Huang, sp.n. (Figure 2)

*Diagnosis. Aphelinus albimaculatus*, **sp.n.** belongs to the subgenus *Aphelinus* (*Aphelinus*) and resembles *A. takecallis*, but can be distinguished from the latter by: metasoma largely pale yellow, both sides of T1–T2 with light brown spots, both sides of T3 with triangular brown spots, T4–T7 largely light brown (*A. takecallis*: base of metasoma yellow, T3–T7 pale brown, third valvula brown); fore wing with 2 lines of 26 setae basal to the linea calva, the basal area with 3 setae (*A. takecallis*: with 1 complete line and 1 incomplete line of 18–20 setae basal to the linea calva, basal cell with 1–2 setae); the coxae of fore leg and mid leg pale brown (*A. takecallis*: yellow), mid leg and hind leg with the tip of femur with a light brown spot (*A. takecallis*: with brown spot).

*Description*. **Female.** Body length 1.04–1.11 mm. **Color.** Head yellow brown, eyes dark red, antenna pale yellow; mesosoma brown; metasoma largely pale yellow, sides of T1 and T2 with light brown spots, sides of T3 with triangular brown spots, T4–T7 largely light brown; wing hyaline; fore leg with the basal 1/2 of coxae brown, the tip pale, femur, tibia pale yellow, tarsus pale brown; mid leg with coxae largely pale brown, the tip of coxae pale yellow, femur, tibia, 1–3 tarsus pale yellow, 4–5 tarsus pale brown; hind leg with coxae brown, except the tip pale, femur, tibia, 1–3 tarsus pale yellow, 4–5 tarsus pale brown, the mid leg and hind leg with the top of femur with slightly light brown spots. **Head.** Head 1.11× as broad as high in frontal view, about as broad as mesosoma; frontovertex 0.39× head width; mandible with two acute teeth; antennae with scape 5.55× as long as broad, pedicel 2.11× as long as broad, F1 trapezoid, F2 1.09× as broad as long, F3 approximately tetragonum, club 2.48× as long as broad. **Mesosoma.** Mesonotum with fine reticulate sculpture, mid lobe of mesoscutum with 35–40 fine setae and 2 pairs of long setae, each side lobe with 1 long and 1 short seta; mesoscutellum with 2 pairs of long setae; mesotibial spur 0.86× mesobasitarsus. **Fore wing.** 2.23× as long as broad; submarginal vein with 6 setae, marginal vein with 9–10 setae along anterior margin, stigmal vein short; 2 lines of 26 setae basal to the linea calva, the basal area with 3 setae; wing disc with dense setae. **Leg.** Tarsal formula 5-5-5. **Metasoma.** 1.57× as long as mesosoma; ovipositor located basally at basal 1/3 of metasoma, 1.28× as long as mesotibia, third valvula 0.42× length of ovipositor.

**Male**. Unknown.

*Host*. Indet. aphids on bamboo.

*Distribution*. China (Beijing).

*Etymology*. The species name is derived from the Latin, *albi* = pale, *maculatus* = spot, referring to the gaster with pale spots.

Material. **Holotype** ♀, No. Y44, **ex.** indet. aphids on bamboo. China: Beijing, Crab Island, 21 October 2011, coll. Zhuhong Wang (FAFU).

**Figure 2:**
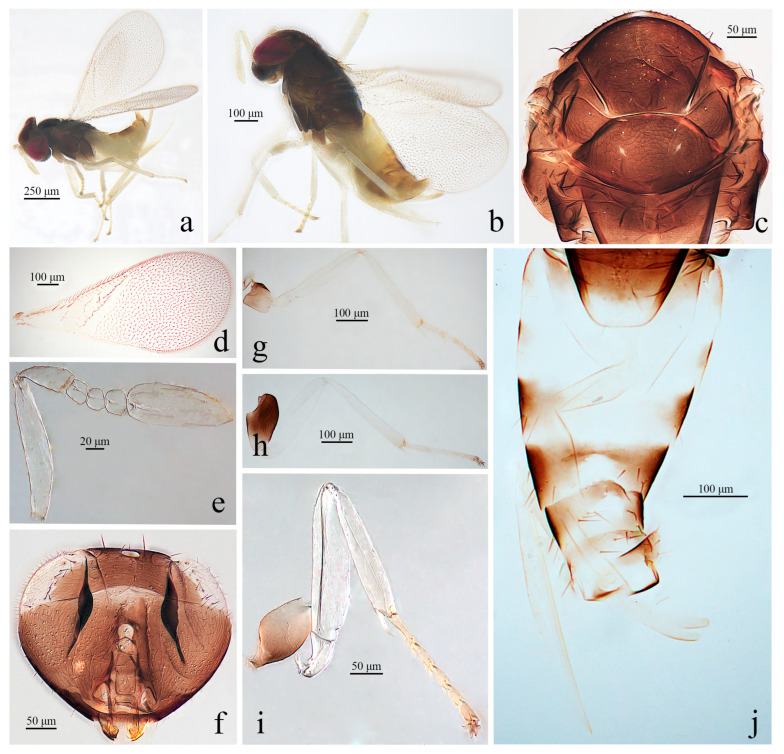
*Aphelinus albimaculatus*, **sp.n**., female. (**a**) Adult in lateral view; (**b**) adult in dorsal view; (**c**) mesosoma; (**d**) fore wing; (**e**) antenna; (**f**) head; (**g**) mid leg; (**h**) hind leg; (**i**) fore leg; (**j**) metasoma.

### 3.2. The Species of the japonicus Group

The *japonicus* group was established by Hayat (1991) [20] and originally included only one species, *Aphelinus japonicus* Ashmead. The new species described below, *Aphelinus varius* Wang & Huang, **sp.n.**, is the second member of this group. The *japonicus* group can be distinguished by: antenna 6-segmented in the female and 5-segmented in the male; body yellow, partly dark brown to black; the numerous setae on mid-lobe not arranged in symmetry; ovipositor short, hypopygium not reaching the apex of the gaster.

Key to species of the *japonicus* group:

Pronotum, mesonotum and most of axillae yellow; scutellum, metanotum and propodeum dark brown to black. digitus of the genitalia with two claspers of the male........................................................................................***A. japonicus***
Pronotum, mesonotum, axillae, scutellum, metanotum and propodeum black. digitus of the genitalia with three claspers of the male....................................................................................***A.varius*, sp.n.**

#### 3.2.1. *Aphelinus japonicus* Ashmead [20,21,29] (Figure 3 and Figure 4)

*Aphelinus japonicus* Ashmead, 1904, Journal of the New York Entomological Society, 12(3): 161. Syntypes 2♀, JAPAN: Atami (USNM); redescription by Hayat, 1991, Entomon, 16(3): 179–181; first description of the male by Hayat, 1994, Entomon, 1994, 19(3–4): 85–89; Chen & Li, 2016, Journal of Northeast Forestry University, 44(11): 100. [20,21,29,30].

*Host*. Indet. aphids on bamboo.

*Distribution*. China, Japan.

Material. 1♀, **ex.** indet. aphids on bamboo, China: Fujian, Fuzhou, Minghou, 1 June 2014, coll. Zhuhong Wang (FAFU).

**Figure 3:**
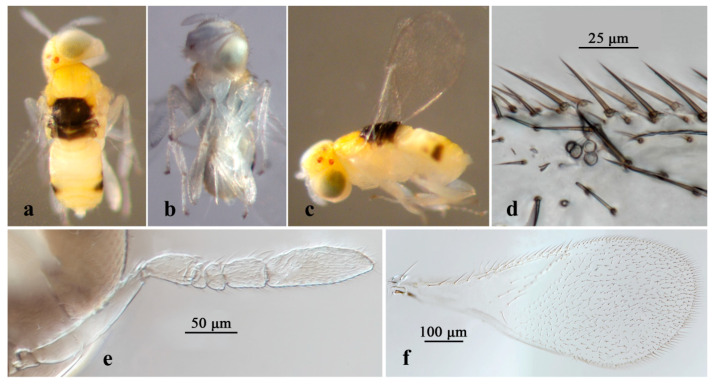
*Aphelinus japonicus*, female. (**a**) Adult in dorsal view; (**b**) adult in ventral view; (**c**) adult in lateral view; (**d**) stigmal vein and sensilla of fore wing; (**e**) antenna; (**f**) fore wing.

Note. *Aphelinus japonicus* Ashmead, a little known species collected from Japan, was redescribed by Hayat (1991) [20]. Hayat (1991) pointed out that *A. japonicus* differs from all the known species of the genus by the partly dark color of the thorax, the numerous setae on mid-lobe not arranged in symmetry, the short ovipositor, and the hypopygium not reaching the apex of the gaster, and regarded *A. japonicus* as a species of a separate group, the *japonicus* group, not placed in the three subgenera recognized by Hayat (1990) [19]. The male of *A. japonicus* was described by Hayat (1994) [21] as having a 3-segmented flagellum and clearly different from all other described species of *Aphelinus* which have a 4-segmented flagellum in both males and females. In China, *A. japonicus* was first recorded by Chen & Li (2016) [30] from the female, and this is the second report of the female.

**Figure 4:**
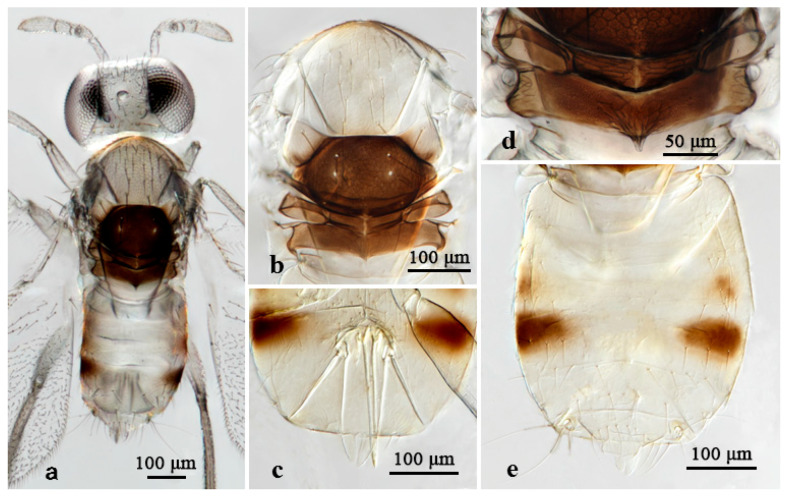
*Aphelinus japonicus*, female. (**a**) Adult in dorsal view; (**b**) mesosoma; (**c**) ovipositor; (**d**) metanotum and propodeum; (**e**) metasoma.

#### 3.2.2. *Aphelinus varius* Wang & Huang, sp.n. (Figure 5 and Figure 6)

*Diagnosis. Aphelinus varius*, **sp.n**. belongs to the *japonicus* group and resembles *A. japonicus*, but can be distinguished from the latter by: mesosoma black (*A. japonicus*: mostly yellow, scutellum, metanotum and propodeum except on both sides black); antennae with scape pale yellow, other yellow brown (*A. japonicus*: antennae white to pale yellow); legs yellow, except last tarsal segments, claw black (*A. japonicus*: legs including coxae white to pale yellow).

*Description.* **Female.** Body length 0.99–1.34 mm. **Color.** Head yellow; eyes yellow with a dark spot; ocellus isosceles triangle; antennae with scape pale yellow, the others yellow brown; mesosomal terga black, mesosomal sterna yellow; metasoma yellow, sides of T2–T4 with black spots; wing hyaline; legs yellow, except last tarsal segments, claw black. **Head**. Head 1.23× as broad as high in frontal view, about as broad as mesosoma; frontovertex 0.35× width of head; mandible with two acute teeth; antennae with scape 5.85× as long as broad, pedicel 2.04× as long as broad, F1 small, nearly trapezoid, 1.5× as broad as long, F2 nearly parallelogram, 1.41× as long as F1, F3 nearly trapezoid, 1.48× as long as combine of F1 and F2, club 2.62× as long as broad and 2.36× as long as F3. **Mesosoma.** Mesosoma and scutellum with fine reticulate sculpture, mid lobe of mesoscutum with 1 pair of long setae and about 25 fine setae, side lobe each with 1 long and 3 fine setae; scutellum with 2 pairs of long setae. **Fore wing**. 2.26× as long as broad; costal cell 1.30× as long as marginal vein; submarginal vein bearing 3 setae, marginal vein bearing 16 setae along the anterior margin; stigmal vein short; one complete line of 15 setae basal to the linea calva and 2 additional setae in angle with marginal vein. **Leg.** Tarsal formula 5-5-5. **Metasoma**. 1.13× as long as mesosoma; ovipositor located basally at basal 1/3 of metasoma, 0.79× as long as mesotibia, third valvula 0.15× length of ovipositor.

**Figure 5:**
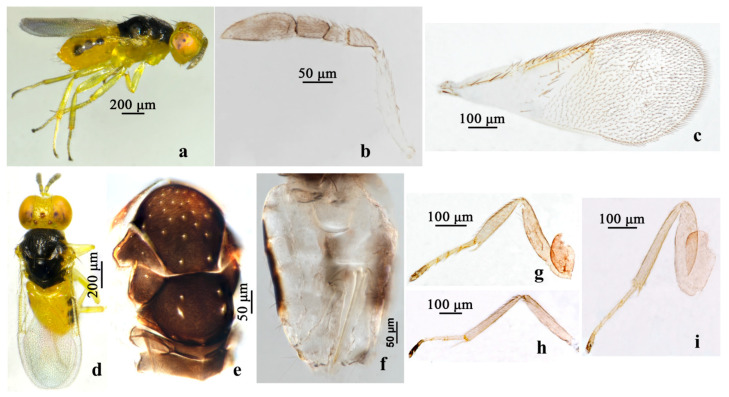
*Aphelinus varius*, **sp.n.**, female. (**a**) Adult in lateral view; (**b**) antenna; (**c**) fore wing; (**d**) adult in dorsal view; (**e**) mesosoma; (**f**) metasoma; (**g**) fore leg; (**h**) mid leg; (**i**) hind leg.

**Figure 6:**
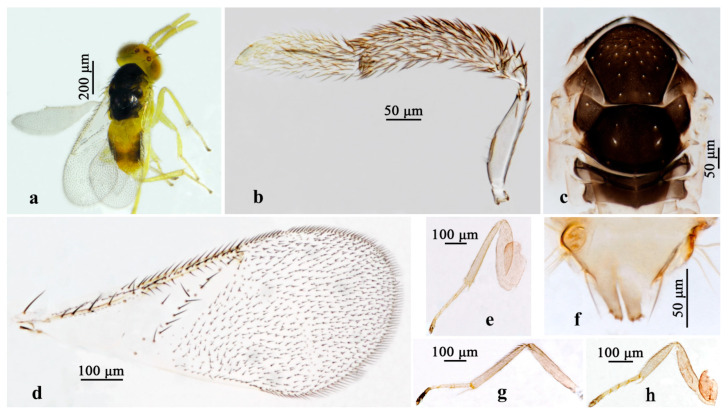
*Aphelinus varius*, **sp.n.**, male. (**a**) Adult in dorsal view; (**b**) antenna; (**c**) mesosoma; (**d**) fore wing; (**e**) fore leg; (**f**) digitus of genitalia; (**g**) mid leg; (**h**) hind leg.

**Male**. Body length 0.97–1.00 mm. Similar to female except: antennae with a 3-segmented flagellum-bearing dense setae, the first segment small and hardly visible, the second long and expand, 3.69× as long as broad, the third shorter and narrower than the second, 3.33× as long as broad, scape 4.11× as long as broad, with a distinct sensillum ventrally; T5 black mostly; digitus of the genitalia with three claspers.

*Host.* Indet. aphids on *Podocarpus macrophyllus* (Thunb.)

*Distribution.* China (Fujian).

*Etymology*. The species name is derived from the Latin, *varius* = varying, referring to the obvious variation of antennal flagellum in the male.

Material. **Holotype** ♀, No.Y40, **ex.** indet. aphids on *Podocarpus macrophyllus* (Thunb.). China: Fujian, Fuzhou, Fujian Agriculture and Forestry University, 10 November 2024, coll. Zhigang Dong and Ye Luo (FAFU); **Paratype** 1♂, same data as holotype (FAFU).

### 3.3. Phylogenetic Analysis

**Figure 7:**
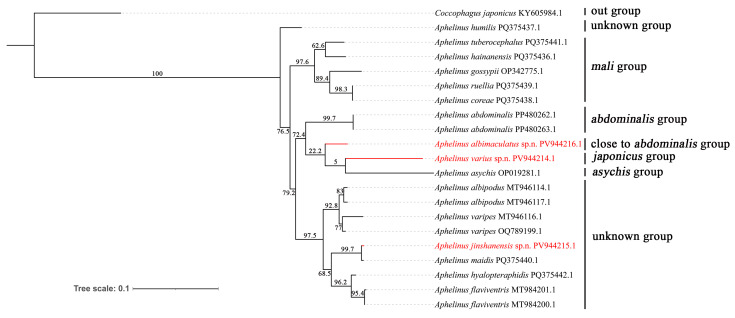
The maximum likelihood (ML) phylogenetic tree based on the *Aphelinus cox1* gene. Bootstrap support values indicated on branches.

Phylogenetic relationships of some species of *Aphelinus* were analyzed based on three newly sequenced *cox1* genes and 17 previously published *cox1* genes from GenBank. One species of *Coccophagus*, *C. japonicus*, was selected as the outgroup. The phylogenetic relationships of some *Aphelinus* species are presented in Figure 7. Maximum likelihood (ML) analysis recovered the tribe *Aphelinus* as monophyletic. However, the bootstrap support along the backbone is generally low, indicating that the phylogenetic relationships between major clades within *Aphelinus* remain unresolved. While some subclades, such as *mali* group, *abdominalis* group, are identifiable, the higher-level branching patterns among these major clades lack strong support. Notably, all species assigned to the same morphological group clustered together in monophyletic clades. Among specific relationships, *Aphelinus jinshanensis*, **sp.n.** formed a strongly supported sister clade (99.7% bootstrap) to *A. maidis*. Similarly, *A. abdominalis* was recovered as the sister group to a clade comprising *A. albimaculatus*, **sp.n.** + (*A. varius*, **sp.n.** + *A. asychis*) with moderate support (72.4% bootstrap). This topological placement indicates *A. albimaculatus*, **sp.n.** is more closely related to the *japonicus* group and *asychis* group (22.2% bootstrap) than to *abdominalis* group, which contrasts with its morphological classification (close to the *abdominalis* group). A precise species–group classification for *A. albimaculatus*, **sp.n.** is expected from future research involving more specimen sampling and genetic analyses. In addition, the proposed sister relationship between *A. varius*, **sp.n.** (*japonicus* group) and *A. asychis* (*asychis* group) received negligible statistical support (5% bootstrap), indicating that this specific relationship remains unresolved.

## 4. Discussions

*Aphelinus* is currently classified into three subgenera: *Aphelinus*, *Indaphelinus* and *Mesidia* [8]. Within the subgenus *Aphelinus*, four species groups are recognized: *abdominalis*, *asychis*, *mali* and *nepalensis* [8]. In this study, we adopt the revised classification of the *mali* group, following Hopper et al. (2012) [31] and Dong et al. (2024) [18], which is characterized mainly by a dark head and body, partly pale metasoma, and fore wing with a complete row of setae basal to the linea calva. Consequently, some species from Hayat (1998) [8] were excluded from our *mali* group concept. This revised *mali* group, within the subgenus *Aphelinus*, was strongly supported as a monophyletic clade in the phylogenetic analysis (Figure 7).

The phylogenetic placement of the three newly described species provides insights into the classification of the genus *Aphelinus*. In the phylogenetic analysis, *A. albimaculatus*, **sp.n.** forms a sister group relationship with the *japonicus* group + *asychis* group, with a support value of only 22.2% (Figure 7) and it is closely related to the *abdominalis* group on the main branch, with a support value of 72.4% (Figure 7). Morphologically, *A. albimaculatus*, **sp.n.** is close to the *abdominalis* group; while there are some morphological distinctions, such as the basal area of fore wing with 3 setae, frontovertex width slightly longer than eyes width, it differs more significantly from the *japonicus* and *asycphis* groups. But the phylogenetic analysis shows that *A*. *albimaculatus*, **sp.n.** is more closely related to the *japonicus* and *asycphis* groups than to the *abdominalis* group, although they belong to the same main clade. Morphological and phylogenetic analyses reveal certain inconsistencies, which may be attributed to the limited genetic data available for genus *Aphelinus* in existing databases, or the inability of a single *cox1* gene to fully resolve the taxonomic position of *A. albimaculatus*, **sp.n.** We will collect more species and obtain additional gene sequences, such as mitochondrial genomes and nuclear genes, to improve phylogenetic resolution. The positions of the other two new species highlight more complex taxonomic relationships. *A. jinshanensis*, **sp.n.**, characterized by its black body, does not cluster with any of the four defined species groups in the subgenus *Aphelinus*. Instead, it forms a clade with eight other species of uncertain group affiliation, a finding corroborated by its distinct morphology. *A. varius*, **sp.n.**, described here as the only second species in the *japonicus* group, underscored the distinctiveness of this group. The *japonicus* group is separated from the three known subgenera by a key autapomorphy: a 3-segmented flagellum in the male, in contrast to a 4-segmented flagellum found in both sexes of all other *Aphelinus* species. This group, along with the brachypterous *asychis* group, represents a lineage with pronounced morphological divergence. The phylogenetic analysis on ML tree tentatively clustered these two groups together, correlating with their shared morphological distinctness. However, the molecular support for this sister–group relationship is notably low (5% bootstrap), indicating this relationship is uncertain. This may be due to the complex evolutionary history reflected in their current subgeneric classification, wherein the *asychis* group belongs to a defined subgenus while the *japonicus* group does not. Meanwhile, this may also be attributed to the insufficient amount of molecular data. No molecular sequences of *A. japonicus* was obtained in this study. In the future, we will continue to collect specimens of this species to acquire its molecular sequences, thereby improving the taxonomic status of this group.

Current molecular phylogenetic analyses within the genus *Aphelinus* are limited by the sparse taxonomic sampling in public databases. GenBank currently contains *cox1* sequence data primarily for the subgenus *Aphelinus*, with no molecular data available for the subgenera *Indaphelinus* and *Mesidia*, or for the critical species *A. japonicus*. Therefore, the molecular data cannot adequately resolve the taxonomic status of the *japonicus* group. In contrast, morphological evidence clearly distinguishes the *japonicus* group from the three established subgenera, particularly through a male-specific autapomorphy (3 -segmented flagellum) that is unique within the genus *Aphelinus*. Therefore, future studies, particularly those incorporating broader molecular sampling, are necessary to determine whether the *japonicus* group warrants elevation to subgeneric rank.

## Figures and Tables

**Table 1:** Primers and cycling conditions.

Primer	Sequence	Cycling Conditions			
LCO1490	5′-GGTCAACAAAATCATAAAGATATTGG-3′	Denaturation	Annealing	Extension	Cycles
HCO2198	5′-TAAACTTCAGGGTGACCAAAAAATCA-3′	94 °C (30 s)	47 °C (30 s)	72 °C (1 min)	35

**Table 2:** The species and GenBank accession numbers used in phylogenetic analysis.

Family/Species	GenBank Accession No.	Source
Aphelinidae		
*Aphelinus abdominalis*	PP480262.1	GenBank
*A. abdominalis*	PP480263.1	GenBank
*A. asychis*	OP019281.1	GenBank
*A. gossypii*	OP342775.1	GenBank
*A. varipes*	OQ789199.1	GenBank
*A. varipes*	MT946116.1	GenBank
*A. coreae*	PQ375438.1	[18]
*A. hainanensis*	PQ375436.1	[18]
*A. humilis*	PQ375437.1	[18]
*A. hyalopteraphidis*	PQ375442.1	[18]
*A. maidis*	PQ375440.1	[18]
*A. ruellia*	PQ375439.1	[18]
*A. tuberocephalus*	PQ375441.1	[18]
*A. albipodus*	MT946114.1	GenBank
*A. albipodus*	MT946117.1	GenBank
*A. flaviventris*	MT984200.1	GenBank
*A. flaviventris*	MT984201.1	GenBank
*A. varius*, **sp.n.**	PV944214.1	Present manuscript
*A. jinshanensis*, **sp.n.**	PV944215.1	Present manuscript
*A. albimaculatus*, **sp.n.**	PV944216.1	Present manuscript
*Coccophagus japonicus*	KY605984.1	GenBank

## Data Availability

Data are contained within the article.
